# FLT3 activating mutations display differential sensitivity to multiple tyrosine kinase inhibitors

**DOI:** 10.18632/oncotarget.14539

**Published:** 2017-01-06

**Authors:** Bao Nguyen, Allen B. Williams, David J. Young, Hayley Ma, Li Li, Mark Levis, Patrick Brown, Donald Small

**Affiliations:** ^1^ Department of Oncology, Johns Hopkins University School of Medicine, Baltimore, MD, USA; ^2^ Department of Pediatrics Johns Hopkins University School of Medicine, Baltimore, MD, USA

**Keywords:** acute myeloid leukemia, mutant FLT3, activation loop, tyrosine kinase inhibitors

## Abstract

-like tyrosine kinase-3 (FLT3) is a receptor tyrosine kinase that normally functions in hematopoietic cell survival, proliferation and differentiation. Constitutively activating mutations of FLT3 map predominately to the juxtamembrane domain (internal tandem duplications; ITD) or the activation loop (AL) of the kinase domain and are detected in about 1/3 of *de novo* acute myeloid leukemia (AML) patients. Small molecule tyrosine kinase inhibitors (TKI) effectively target FLT3/ITD mutations, but some activating mutations, particularly those on the AL, are relatively resistant to many FLT3 TKI. We reproduced many of the AL or other non-ITD activating mutations and tested 13 FLT3 TKI for their activity against these and wild-type FLT3. All 13 TKI tested inhibited BaF3/ITD cell proliferation in a concentration-dependent manner as reported, but most TKI exhibited a wide range of differential activity against AL and other point mutants. Western blotting results examining inhibition of FLT3 autophosphorylation and signaling pathways indicate that many AL mutations reduce TKI binding. Most FLT3 TKI effectively target wild-type FLT3 signaling. As a demonstration of this differential activity, treatment of BaF3 D835Y cells transplanted in BALB/c mice with sorafenib showed no effect *in vivo* against this mutant whereas lestaurtinib proved effective at reducing disease burden. Thus, while FLT3 TKI have been selected based on their ability to inhibit FLT3/ITD, the selection of appropriate TKI for AML patients with FLT3 AL and other activating point mutations requires personalized consideration.

## INTRODUCTION

Fms-like tyrosine kinase 3 (FLT3) is a member of the class III family of receptor tyrosine kinases, including platelet-derived growth factor receptors α and β, c-kit and fms. [1, 2] Their general structure consists of five extracellular immunoglobin-like domains, a single transmembrane domain, a short juxtamembrane region and an interrupted kinase domain. FLT3 expression is limited primarily to primitive hematopoietic stem/progenitor cells and dendritic cells. It plays roles in the processes of differentiation, survival and proliferation. The kinase activity of FLT3 is normally stimulated upon binding of FLT3 ligand (FL), which leads to receptor dimerization, autophosphorylation, and phosphorylation of other signaling proteins. [3, 4] This, in turn, leads to activation of downstream signaling cascades, including signal transducers of activation and transcription (STATs), mitogen-activated protein kinases (MAP kinases) and phosphatidyl inositol-3 kinase/AKT pathways. Mutation of FLT3 induces constitutive kinase activation which is independent of ligand and occurs in about one-third of AML cases. [5, 6] About 20-25% of AML patients express FLT3 mutations as in-frame internal tandem duplications (ITDs) of varying length in the juxtamembrane domain. An additional 7-10% of patients harbor point mutations of the kinase domain activation loop (AL) or other areas. Point mutations within the juxtamembrane domain of FLT3 have also been detected and represent a rarer class of activating mutations. [7, 8] The crystal structure of FLT3 shows that the juxtamembrane domain makes contact with the activation loop and maintains FLT3 in an autoinhibited state. [9] Mutations in the juxtamembrane domain or in the activation loop destabilize the inhibitory conformation and lead to constitutive FLT3 activation. Transfection of plasmids containing either type of mutation into cytokine-dependent hematopoietic cell lines transforms cells to growth factor independence. [10] Activating mutations of FLT3 are also observed at lower frequencies in acute lymphoblastic leukemia (ALL), [11, 12] myelodysplastic syndrome (MDS) [13] and mixed-lineage leukemia (MLL) rearranged infant leukemias. [14, 15]

Kinase domain mutations are primarily restricted to the carboxy terminal portion of the catalytic domain, predominately at residues D835 and I836 on the activation loop. [6, 16, 17] The crystal structures of related tyrosine kinases indicate that the activation loop acts as a flexible gate to allow access of ATP and substrate to the nucleotide-binding site when the molecule assumes the “open” or active conformation. [9, 18–20] In the “closed” or autoinhibited conformation, the FLT3 activation loop swings inward and binds to D811, thus preventing ATP binding in this pocket. [9] Mutation of critical residues in the kinase domain destabilize the autoinhibited FLT3 structure, shifting the equilibrium towards the open conformation, leading to constitutive kinase activation in the absence of FLT3 ligand binding. While all FLT3 activating kinase domain mutations provide growth and survival advantages, there appear to be signaling differences compared to ITD mutations. [21, 22] Moreover, amongst the kinase domain mutations, there are wide differences in response to FLT3 tyrosine kinase inhibitors (TKI). [23, 24] All TKI described as FLT3 inhibitors target FLT3/ITD mutations, while some of these TKI show limited activity against wild-type FLT3, and most exhibit significantly reduced potency at inhibiting some of the AL and other point mutations. D835Y is the most frequently observed kinase domain mutation in AML patients, constituting ~50% of all FLT3 AL missense mutations. [25] Other substitution mutations detected less frequently at D835 include valine, histidine, glycine, and asparagine substitutions as well as aspartic acid codon deletion. [6, 16, 17, 26] The I836 codon also undergoes deletion and/or mutation to threonine, serine or leucine with or without an insertion. [6, 16] Several of the additional mutations dispersed on the activation loop or in the kinase domain have been documented in patients at D593D [27], S840 [28], N841 [29] and Y842 [30] .

In this study, BaF3 cells transfected with different FLT3 activating mutations reported in patients were tested against a panel of 13 compounds characterized as FLT3 inhibitors. Five of them are currently in clinical trials for treatment of acute myeloid leukemia including sorafenib [31], quizartinib [32], midostaurin [33], crenolanib [34] and ponatinib [35] . Another FLT3 inhibitor, TTT-3002, demonstrated high potency in FLT3/ITD and FLT3/D835 mutated leukemia cell lines and patient samples in preclinical studies [36, 37] . A wide range of responses were noted to each mutation. The results show that most inhibitors are not effective against some activating mutations, or substantially higher drug concentrations are required to inhibit proliferation of cells bearing these particular mutations, concentrations unlikely to be reached in these patients. The data presented here indicate that a number of activating mutations render FLT3 insensitive to some TKI, and determination of the particular mutation expressed should be undertaken when deciding upon a FLT3 TKI regimen for AML patients with AL and other non-ITD point mutations.

## RESULTS

### Differential sensitivity of FLT3 AL mutants to FLT3 TKI growth inhibition

**Figure 1:**
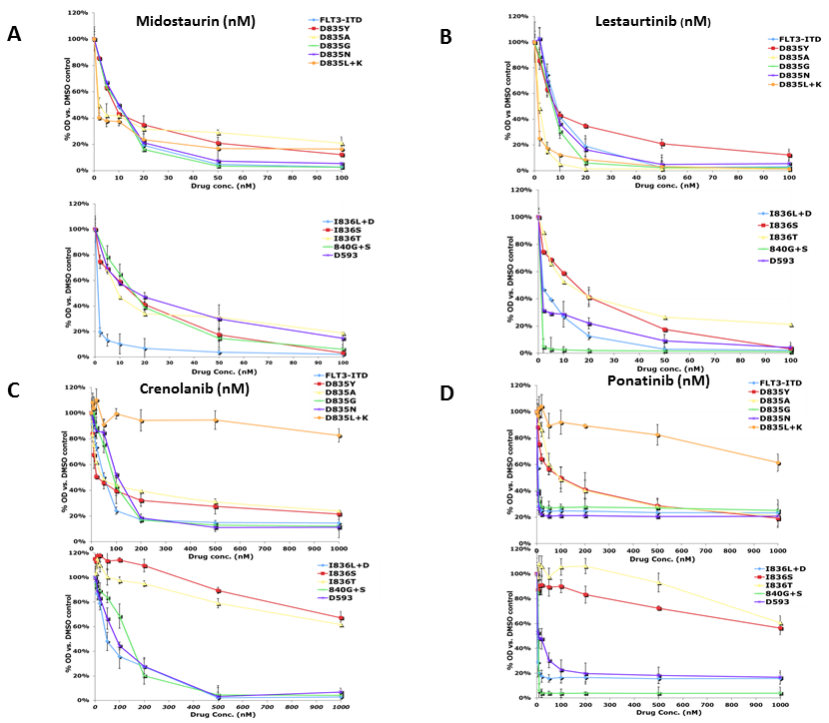
Growth of AL mutants in the presence of FLT3 TKI The BaF3 AL mutants were grown in the absence of IL-3 and analyzed by treatment in increasing concentrations of FLT3 TKI for 48 hours, after which growth inhibition was assessed using the MTT assay. Means are representative of at least three independent experiments.

The FLT3 AL mutations selected for use in this study were all originally isolated from AML patients and transfection of each into BaF3 cells led to IL-3 independent growth. Representative clones were obtained following limiting dilution and their responses to FLT3 TKI were measured by MTT (growth/survival) assay. Each of the 13 FLT3 TKI tested inhibited proliferation of BaF3/ITD mutant cells in a concentration-dependent manner, albeit with widely varying potencies (Table 1 and Figure 1). Lestaurtinib, midostaurin and TTT-3002 inhibited proliferation in all FLT3 AL mutants with generally similar or sometimes even greater potency compared to the FLT3/ITD cells. In contrast, 10 of the 13 TKI tested displayed a range of activity against FLT3 AL mutants. Multiple FLT3 TKI were active against the D835G, D835N, I836T and 840G+S mutants, with IC_50_ values within 10-fold of the values for inhibiting the FLT3/ITD mutant (midostaurin, lestaurtinib, linifanib, quizartinib, sutinib, sorafenib, TTT-3002 and KW2449). However, at least 7 of the 13 FLT3 TKI tested had little or no activity against the D593D, D835Y, D835A, D835L+K, I836L+D and I836S mutants (AG1295, AGS324, linifanib, quizartinib, sunitinib, sorafenib and R406). For those 7 FLT3 TKI the IC_50_ was not reached over the concentration range tested.

**Table 1:** Proliferation IC50 (nM) of FLT3 TKI against AL mutants

FLT3 mutants mut	Midostaurin	Lestaurtinib	AG1295	AGS324	Linifanib	Quizartinib	Sunitinib	Sorafenib	TTT-3002	R406	KW2449	Crenolanib	Ponatinib
**FLT3-ITD**	**9.3**	**8.6**	**297.9**	**65.3**	**2.4**	**1.2**	**5.4**	**18.5**	**<1.0**	**165.6**	**41.0**	**57.0**	**<1.0**
**D835Y**	**10.0**	**9.8**	**>3000**	**>3000**	**>100**	**>100**	**>100**	**>2000**	**4.1**	**>200**	**>200**	**58.0**	**92.0**
**D835A**	**5.0**	**5.0**	**1972.0**	**>200**	**>200**	**>200**	**>200**	**>200.0**	**1.3**	**>200**	**62.0**	**78.1**	**148.2**
**D835G**	**7.9**	**6.0**	**423.0**	**60.1**	**8.6**	**9.0**	**48.1**	**34.5**	**<1.0**	**141.0**	**56.0**	**110**	**<1.0**
**D835N**	**8.4**	**7.6**	**411.1**	**100**	**10.3**	**7.2**	**46.4**	**31.6**	**<1.0**	**153.0**	**58.3**	**80.0**	**<1.0**
**D835L+K**	**1.0**	**2.1**	**>3000**	**>3000**	**>200**	**>200**	**>200**	**>200**	**6.7**	**>200**	**>200**	**>1000**	**>1000**
**I836L+D**	**5.2**	**2.1**	**>3000**	**>3000**	**>200**	**>200**	**>200**	**>200**	**11.7**	**>200**	**>200**	**50.0**	**<1.0**
**I836S**	**10.2**	**15.0**	**>3000**	**>200**	**>200**	**>200**	**>200**	**10.2**	**9.0**	**>200**	**>200**	**>1000**	**>1000**
**I836T**	**10.0**	**13.4**	**>3000**	**>3000**	**5.3**	**6.8**	**6.0**	**48.2**	**9.7**	**152.0**	**31.9**	**>1000**	**>1000**
**840G+S**	**12.0**	**8.0**	**500.0**	**50.0**	**2.1**	**2.5**	**4.0**	**5.0**	**<1.0**	**>200**	**35.0**	**83.0**	**<1.0**
**D593D**	**2.4**	**8.0**	**>3000**	**>200**	**>200**	**>200**	**>200**	**>200.0**	**2.8**	**>200**	**>200**	**70.0**	**10.7**

Only 4 of the FLT3 TKI tested in this study inhibited growth of the most frequently seen clinical FLT3 AL mutant D835Y. Midostaurin, lestaurtinib, TTT-3002, and crenolanib inhibited growth of this mutant at concentrations that were at least within 20-fold the concentrations effective against the FLT3/ITD mutant, whereas the other inhibitors were largely ineffective at the FLT3/ITD inhibitory concentrations. Although ponatinib is a potent inhibitor of the FLT3/ITD cells with an IC_50_ below 1 nM, the D835Y mutation shifted the IC_50_ to 92 nM (Table 1). The IC_50_ values for the rest of the inhibitors evaluated against the D835Y mutation were beyond their respective testing ranges in this study. These findings are consistent with clinical data showing that treatment of FLT3/ITD positive AML patients with monotherapy with some FLT3 TKI has frequently given rise to blasts that express D835Y, rendering the cells insensitive to further therapy. [38] Midostaurin, lestaurtinib, TTT-3002, and crenolanib were also the only FLT3 TKI in this study that inhibited growth of the D593D mutant at IC_50_ levels close to those observed in the FLT3/ITD mutant cells.

The FLT3 TKI could be ranked based on the number of mutants they inhibited, with midostaurin, lestaurtinib, and TTT-3002 inhibiting growth of all mutants in the panel. Crenolanib and ponatinib inhibited all but 3 of the mutants (D835L+K, I836T, and I836I), although it should be noted that relatively high concentrations of ponatinib were required to inhibit proliferation in D835Y and D835A mutants. Sorafenib and KW2449 inhibited growth of all but 5 mutants (D835Y, D835L+K, I836L+D, D593D and D835A for sorafenib; D835Y, D835L+K, I836L+D, D593D and I836S for KW2449). Linifanib, quizartinib, and sunitinib each inhibited all but 6 of the mutants, whereas AGS324, AG1295, and R406 were ineffective against 7 of the panel members. These results indicate that treatment of AML patients using some FLT3 TKI would be more effective against a greater spectrum of FLT3 AL mutations than other FLT3 inhibitors.

Proliferation half maximal inhibitory concentration (IC_50_) at 48 h by MTT assay. The cells were grown in the absence of IL3 and analyzed by treatment in increasing concentrations of FLT3 TKI for 48 hours, after which growth inhibition was assessed using the MTT assay. Each experiment was performed at least three times and representative results are shown.

### Uniform sensitivity of FLT3 AL mutants to lestaurtinib, midostaurin and TTT-3002

**Figure 2:**
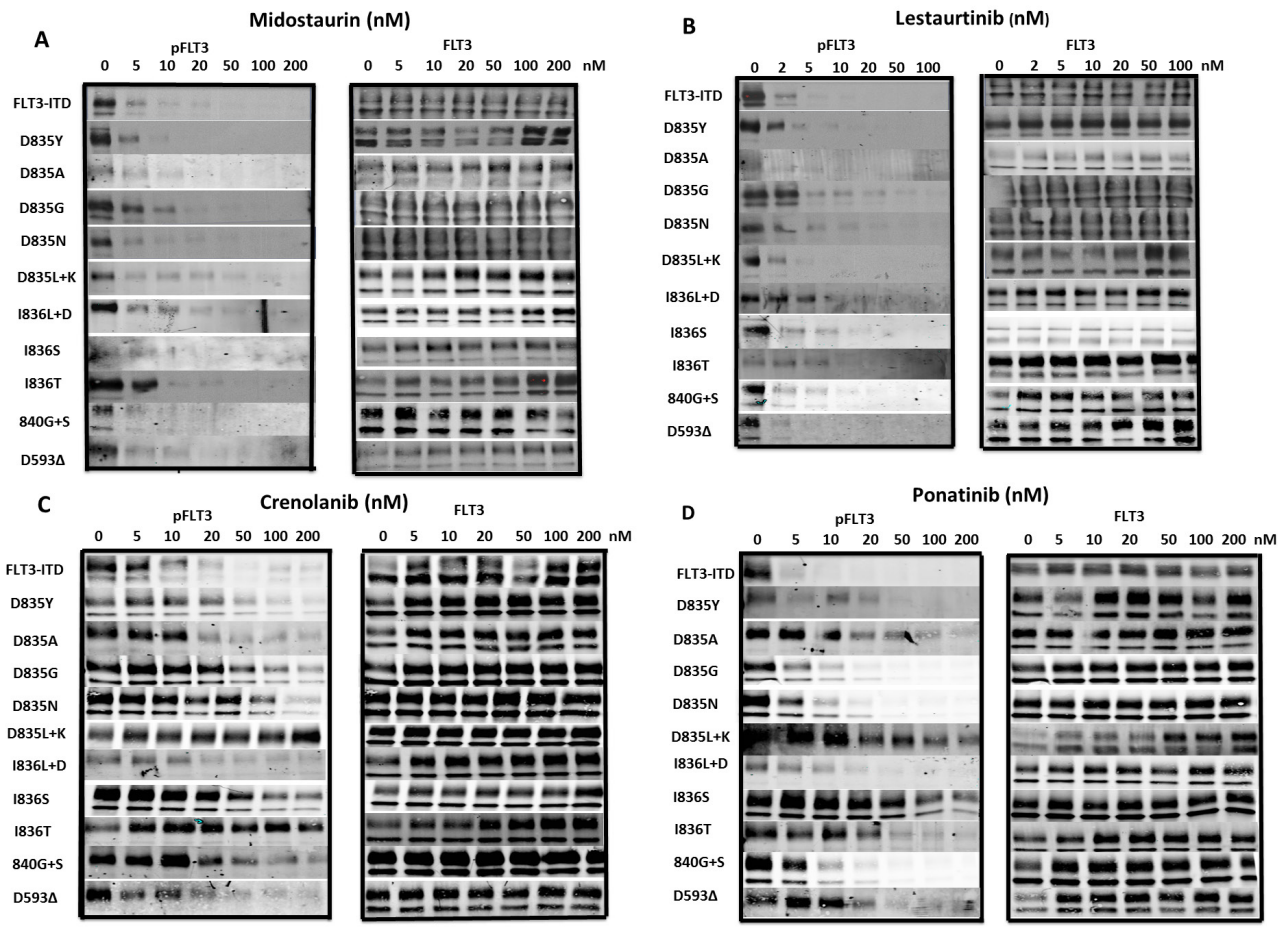
Inhibition of FLT3 AL mutant phosphorylation by FLT3 TKI BaF3 cells expressing FLT3 AL mutations were treated with either **a**. midostaurin, **b**. lestaurtinib, **c**. crenolanib or **d**. ponatinib at the indicated concentrations for 1 h at 37°C followed by immunoprecipitation for FLT3, SDS-PAGE and WB for FLT3 and phospho-FLT3 (PFLT3). Each experiment was performed at least twice and representative results are shown.

Of the 13 FLT3 TKI tested, only lestaurtinib, midostaurin and TTT-3002 inhibited proliferation in all FLT3 AL mutants tested. Lestaurtinib and midostaurin underwent phase 3 clinical trials for FLT3 mutant AML. To that end, we tested the ability of lestaurtinib and midostaurin to inhibit FLT3 autophosphorylation of each FLT3 AL mutant. Midostaurin (Figure 2a) and lestaurtinib (Figure 2b) both inhibited FLT3 autophosphorylation of all FLT3 AL mutants with IC_50_ values of 5 nM or lower. The Western blotting results examining FLT3 inhibition by lestaurtinib and midostaurin for each FLT3 AL mutant correlate well with the MTT data.

### Crenolanib and ponatinib have better activity against FLT3/D835 point mutants than the other FLT3 AL mutants

Crenolanib and ponatinib are both in clinical trials for the treatment of AML patients with FLT3 mutations. One study recently demonstrated that crenolanib displays activity against several of the FLT3 AL mutants as well as FLT3/ITD-TKD dual mutants. [39] We observed that crenolanib inhibited FLT3 autophosporylation of D835Y, D835A, D835G, D835N, I836L+D, 840G+S and D593D mutants with IC_50_s of 36, 20, 28, 30, 15, 104 and 12nM, respectively (Figure 1c). Ponatinib inhibited FLT3 autophosphorylation of D835Y, D835A, D835G, D835N, I836L+D, 840G+S and D593D mutants with IC_50_ values of 5 nM or less (Figure 1d). However, crenolanib and ponatinib were not nearly as effective at inhibiting phosphorylation of D835L+K, I836S and I836T FLT3 mutants, with significant autophosphorylation still observable even at concentrations of 200 nM (Figure 2c, 2d).

### Effect of FLT3 TKI on wild-type FLT3 in the presence of FLT3 Ligand

**Figure 3:**
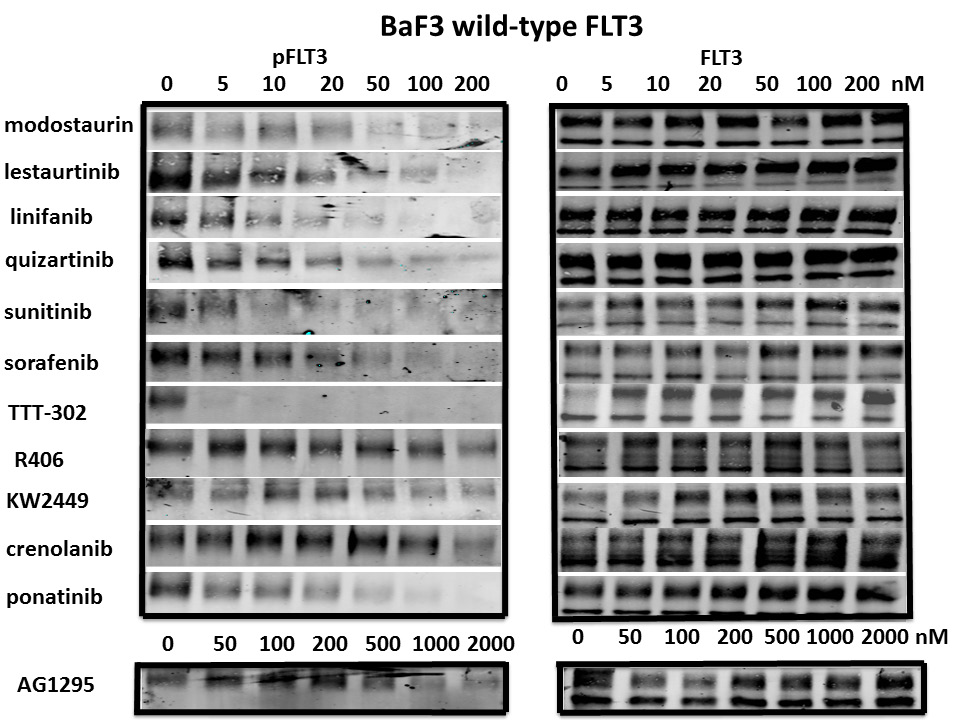
Inhibition of wild-type FLT3 signaling pathways by FLT3 TKI BaF3/WT-FLT3 cells were treated with FLT3 TKI at the indicated concentrations for 1 h at 37°C then stimulated with 25ng/ml of human FLT3 ligand for 15 minutes, followed by immunoprecipitation for FLT3, SDS-PAGE and WB for FLT3 and phospho-FLT3 (PFLT3). Each experiment was performed at least twice and representative results are shown.

Infant ALL, B-lineage ALL and AML samples with the highest levels of wild-type FLT3 expression often show constitutive FLT3 phosphorylation as a result of FL expression by autocrine or paracrine mechanisms. [40] These samples are often sensitive to FLT3 TKI, arguing that wild-type FLT3 signaling is important in these cells. Thus targeting of wild-type, non-mutant FLT3 may be important for this subset of ALL and AML patients. Thus it is important to study the effect of FLT3 TKI on wild-type FLT3 signaling as well. We determined the activity of FLT3 TKI against BaF3/wild-type FLT3 expressing cells that were stimulated with FL (Figure 3). Of the 12 FLT3 TKI tested, the majority of them inhibited FLT3 autophosphorylation of wild-type FLT3 with IC_50_ values of 20 nM or lower. Only Crenolanib, R406 and AG1295 were not nearly as effective at inhibiting wild-type FLT3 phosphorylation compared to FLT3/ITD ( IC_50_ values of 200 nM, 200 nM and 500 nM). Similar results were seen whether cells were incubated with FL first followed by FLT3 TKI treatment or FLT3 TKI followed by FL stimulation.

### Effect of FLT3 TKI on ITD *versus* D835Y signaling pathways

**Figure 4:**
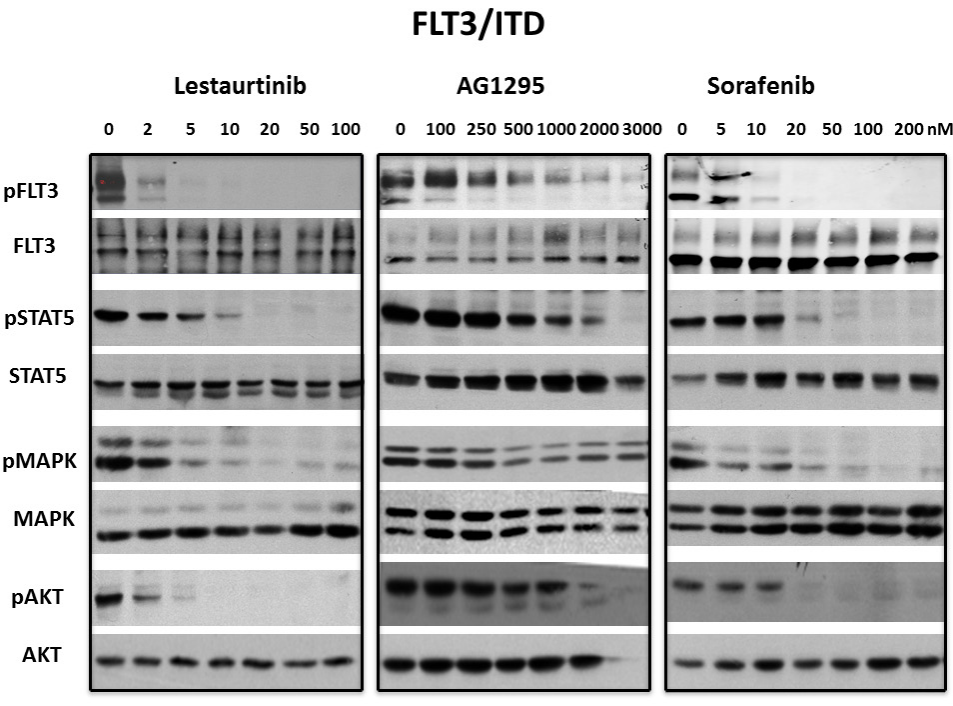
Inhibition of FLT3/ITD signaling pathways by FLT3 TKI BaF3/ITD cells were treated with lestaurtinib, AG1295 or sorafenib at the indicated concentrations for 1 h. Inhibition of signaling pathways by WB was evaluated in whole cell lysates using antibodies described in Materials and Methods and visualizing bands using enhanced chemiluminescence. Each experiment was repeated at least three times and representative results are shown.

**Figure 5:**
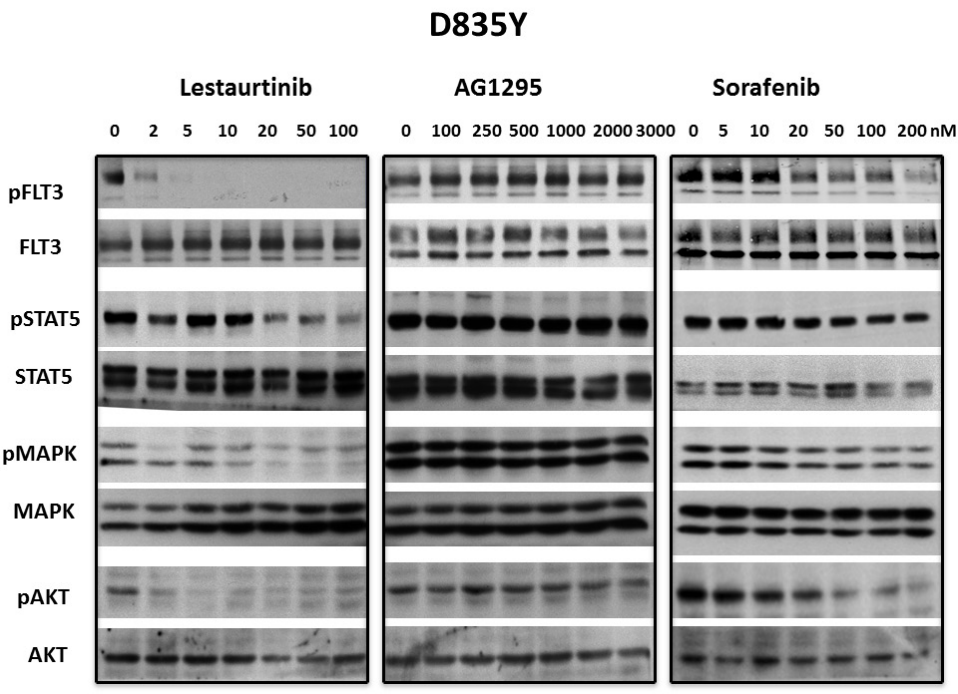
Inhibition of FLT3 D835Y signaling pathways by FLT3 TKI BaF3 FLT3 AL mutant cells were treated with lestaurtinib, AG1295 or sorafenib at the indicated concentrations for 1 h. Inhibition of signaling pathways by WB was evaluated in whole cell lysates using antibodies described in Materials and Methods and visualizing bands using enhanced chemiluminescence. Each experiment was repeated at least three times and representative results are shown.

The STAT5, PI-3 kinase/AKT and Ras/Map kinase pathways are activated by FLT3 and are important in cell survival and proliferation in cells that are dependent on FLT3 activity. However, there are also extrinsic mechanisms independent of FLT3, capable of maintaining signaling pathways downstream of FLT3 despite the presence of inhibitory FLT3 TKI levels. [41] In addition, off-target effects of some TKI that cause inhibition of downstream pathways might cause inhibition of growth despite lack of inhibition against a FLT3 mutant. To determine whether inhibition of FLT3 signaling pathways correlated with inhibition of FLT3 autophosphorylation, 3 FLT3 TKI representing different classes of inhibitors were tested against the FLT3/ITD and the FLT3 D835Y mutants. Treatement with lestaurtinib, sorafenib and AG1295 all inhibited FLT3 autophosphorylation as well as phosphorylation of STAT5, AKT and Map kinase in FLT3/ITD cells in a concentration-dependent manner (Figure 4). In FLT3 D835Y cells, lestaurtinib inhibited FLT3 autophosphorylation with an IC_50_ < 2 nM which resulted in termination of signaling through STAT5, AKT and MAP kinase pathways (Figure 5). In contrast, even the highest concentrations of sorafenib and AG1295 tested showed markedly reduced or absent inhibition of FLT3 autophosphorylation and a subsequent lack of inhibitory activity on phosphorylation of STAT5, AKT and MAP kinase. Thus, for the 3 FLT3 TKI tested against the FLT3/ITD and the FLT3 D835Y mutants, there was a good correlation between inhibition of FLT3 phosphorylation and inhibition of FLT3 dependent downstream signaling pathways.

### Effect of FLT3 TKI on engraftment levels of FLT3 D835Y mutant cells in BALB/c mice

**Figure 6:**
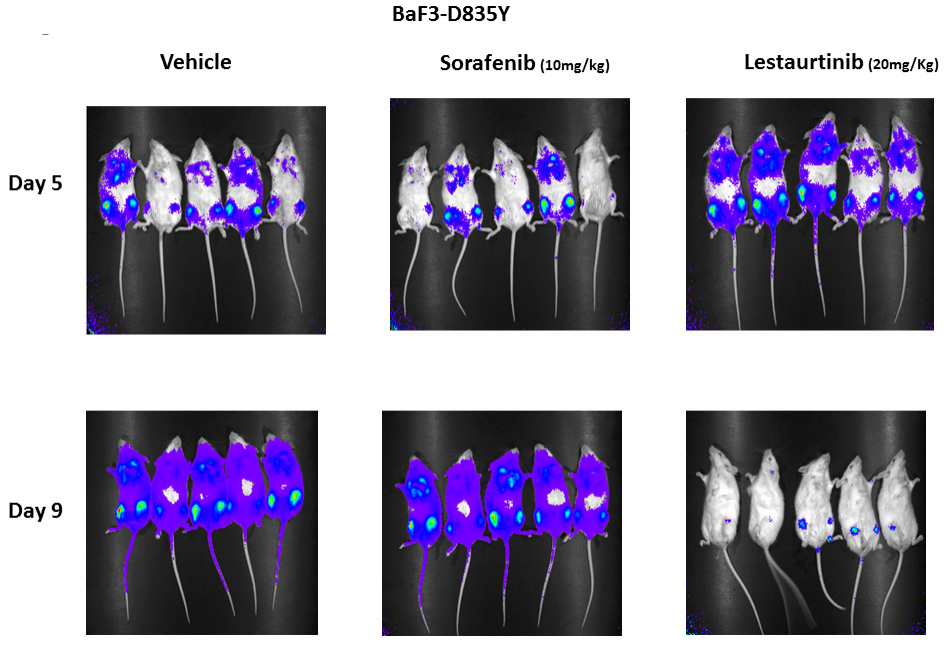
Effect of FLT3 TKI on progression of BaF3 D835Y cells engrafted in syngeneic BALB/c mice BaF3 D835Y cells (2 × 10^6^) transfected with a luciferase construct were transplanted into three cohorts of BALB/c mice *via* tail vein injection with 5 mice per group. On day 5 following transplantation, the level of engraftment was assessed by imaging mice for bioluminescence on an IVIS Spectrum imager. Starting on day 5, mice were then treated twice daily by vehicle, subcutaneous lestaurtinib (20 mg/kg) or once daily sorafenib (10 mg/kg) by oral gavage until day 9, at which point mice were again imaged. This experiment was repeated three times.

Lestaurtinib and sorafenib both inhibit proliferation driven by signaling events in FLT3/ITD cells *in vitro*, but they have divergent effects on the FLT3 AL mutants. To assess their activity *in vivo*, BaF3 FLT3 D835Y cells were transplanted into syngeneic mice and were treated with lestaurtinib or sorafenib. Because the cells were stably transfected with luciferase, disease progression could be monitored by visualizing bioluminescence upon intraperitoneal injection of luciferin. Typically, these cells home to the bone marrow and spread to the spleen and peripheral blood before mice become moribund. By day 5 FLT3 D835Y cells were well engrafted in the mice. By day 9, 4 days after treatment was instituted, the sorafenib and vehicle treated mice demonstrated clear progression of leukemia. In contrast, leukemic burden was greatly reduced in mice treated with lestaurtinib, with only a small amount of D835Y cells detected in the bone marrow (Figure 6).

## DISCUSSION

Nearly half of acute myeloid leukemia patients treated with chemotherapy have a favorable outcome, but those who present with a FLT3/ITD mutation have a worse prognosis. [42–44] Preclinical and clinical evidence suggest that the addition of a FLT3 TKI to chemotherapy is synergistic and may lead to improved efficacy for those patients. [45] FLT3 AL mutations also constitutively activate FLT3 kinase activity and subsequent downstream signaling pathways that lead to transformation and cytokine independence, but do not appear to result in worse prognosis than non-FLT3 mutant AML patients. The addition of a FLT3 TKI to the therapy of FLT3 AL mutant patients might still further improve their outcome and appeared to do so in a recently reported trial of midostaurin. [46] Unfortunately, many FLT3 AL mutations fail to respond to many of the FLT3 TKI that have been selected for by their potency against FLT3 ITD mutations. [23, 24] Treatment of a FLT3 AL mutant patient with these FLT3 TKI would fail to inhibit FLT3 kinase activity and thus have no potential to contribute to the elimination of leukemic blasts. Thus, in this era of personalized medicine, it is important to know the exact FLT3 AL mutation each patient carries in order to be able to pick the most appropriate FLT3 TKI to use for that patient. This is also important during therapy for a patient with a FLT3 ITD mutation because the FLT3 AL mutations appear to arise frequently through selection in these patients under the pressure of FLT3 TKI therapy.

Most of the clinically available FLT3 TKI display reduced activity against at least some of the FLT3 AL mutants. In this study, only lestaurtinib, midostaurin and TTT-3002 appear to be universally effective against this type of mutation. Treatment with FLT3 TKIs other than lestaurtinib, midostaurin, or TTT-3002 therefore, could prove problematic if patients present with, or are selected for during treatment, one of the FLT3 AL mutations. With the increasing number of FLT3 TKI being developed clinically, it is important to determine the spectrum of FLT3 mutants that would respond to or be resistant to each TKI. [47, 48] For example, sorafenib and quizartinib have both demonstrated some efficacy in reducing peripheral blasts in FLT3 ITD mutant AML patients and bone marrow blasts in some cases, but the data from this study indicate that neither of these inhibitors would be effective against blasts harboring FLT3 mutations of D835Y, D835A, D835L+K or I836L+D. [49–53] In addition, the D835G and D835N mutations caused ~10-fold elevation in the IC_50_ for sunitinib that would likely preclude its use against these mutants.

We know that in infant ALL and in AML with the highest level of wild-type FLT3 expression, FLT3 is constitutively phosphorylated and the cells are sensitive to FLT3 TKI. Therefore, studying the effect of FLT3 TKI on wild-type FLT3 signaling is important. With the exception of one of the TKI (AG1295) tested in this study, all of the others showed a high level of activity against wild-type FLT3 signaling. Thus FLT3 TKIs might prove effective for upfront or relapsed therapy for these patients.

TKI are generally categorized as type I or type II inhibitors. [54] Type I inhibitors directly compete with ATP binding to the nucleotide-binding site and make contact with the tyrosine kinase hinge region. Crystal structures of kinases in complex with inhibitors indicate that type I TKI bind target when the phosphorylated kinase activation loop assumes either the active unfolded “open” conformation or the inactive closed conformation. [55] Type II inhibitors indirectly compete with ATP by binding a hydrophobic allosteric site adjacent to the nucleotide-binding site in addition to the nucleotide-binding site. These inhibitors only bind the target when the activation loop assumes the “closed” conformation. Dasatinib is an example of a type I inhibitor that binds BCR-ABL while imatinib and nilotinib are examples of type II inhibitors. [56] FLT3 mutations lead to constitutive activation by two different mechanisms: ITD mutations that relieve the negative regulatory influence of the juxtamembrane domain on the catalytic domain and the AL mutants that directly destabilize the activation loop. [9, 57] There are suggestions that FLT3 TKI also fall into the category of type I or type II inhibitors, but the biological data from this study argue that this categorization, while technically accurate, is likely an oversimplification. Midostaurin (and lestaurtinib) is thought to be a type I inhibitor whereas sorafenib and many other FLT3 TKI are thought to belong to the type II class. [58, 59] Since all FLT3 TKI inhibit ITD mutations but display variable activity against kinase domain mutants, the assertion that type II FLT3 TKI are ineffective against some AL mutations because the kinase is shifted toward the active conformation seems inadequate as it should then hold true for all FLT3 AL mutations. Instead, it is more probable that the activation loop of specific FLT3 AL mutants adopts a conformation that prohibits the DFG residues from forming hydrogen bonds that are characteristic of binding with type II inhibitors, thereby reducing drug affinity. The phenylalanine in the DFG region of activated kinases has been shown to move more than 10 Å from its position in the inhibited conformation. [54] Almost certainly, different substitutions on the activation loop will have varying effects on the position of the DFG residues with subsequently varying effects on the ability of type II FLT3 TKI to tightly bind these mutants. An alternative explanation as to why type II inhibitors might not be effective against FLT3 AL mutants could be that some FLT3 AL mutations don't fully transition to the inactive conformation seen in the autoinhibited form where the activation loop is completely folded between the N and C kinase lobes.

The number of FLT3 activating kinase domain mutations continues to grow and with that the need to define the spectrum of activity of each FLT3 TKI against these mutations. In this report, we demonstrated that TTT-3002, lestaurtinib and midostaurin are fairly effective against the known FLT3 AL mutants reported to date. Despite the development of subsequent generations of FLT3 TKI, many would not be effective in patients that express a FLT3 AL mutation. Thus, there is still a need to identify FLT3 TKI that can target multiple FLT3 activating mutations, both FLT3 ITD and FLT3 AL types, in order to improve upfront AML patient responses and to reduce the incidence of selection of FLT3 AL resistance mutations.

## MATERIALS AND METHODS

### Reagents and antibodies

All compounds were dissolved in dimethyl sulfoxide (DMSO) at stock concentrations of 10 mM and stored at -80°C. Lestaurtinib, quizartinib, midostaurin,, ponatinib, sunitinib and sorafenib were purchased from LCLabs (Ontario, Canada). Linifanib, crenolanib and R406 were purchased from Selleckchem (Houston, Texas, USA). KW2449 was from Kyowa Hakko Kirin Co., Ltd. (Tokyo, Japan). AG1295 and AGS324 were provided by Aviv Gazit (The Hebrew University, Israel). TTT-3002 was a generous gift of TauTaTis, Inc. (San Diego, CA). The final DMSO concentration was maintained at < 0.2% in cell suspension for each drug concentration and control group in all assays. Recombinant human interleukin-3 (IL-3) and human FLT3 ligand (FL) were purchased from Pepro Tech, Inc. (Rocky Hill, NJ, USA). The FLT3 S-18 antibody (#SC-480) was from Santa Cruz Biotechnology (Santa Cruz, CA, USA), 4G10 phosphotyrosine mouse monoclonal antibody and recombinant protein A-agarose were from Upstate Biotechnology (Lake Placid, NY, USA), and CD135-phycoerythrin (PE) conjugated antibody (#558996) was from BD Pharmingen (San Jose, CA, USA). AlexaFluor 680 goat anti-mouse (# A21058) and Daylight 800 goat anti-rabbit antibodies (#611-145-122) were purchased from Invitrogen Corporation (Grand Island, NY, USA) and Rockland Immunochemicals Inc. (Gilbertsville, PA, USA) respectively. PhosphoMAP kinase (#9101), phosphoSTAT5 (#9351), phosphoAKT (#9271), MAP kinase (#9102), STAT5 (#9363) and AKT (#9272) antibodies were from Cell Signaling Technologies, Inc. (Beverly, MA, USA). Goat anti-mouse (#NA931) and goat anti-rabbit (#NA934) horseradish peroxidase antibodies and the enhanced chemiluminescence kit were from Amersham Biosciences (Arlington Heights, IL, USA).

### DNA constructs and cells

BaF3 cells were grown cultured at 37°C in 5% CO_2_ in RPMI medium (Invitrogen Corporation) with 10% fetal bovine serum (Gemini Bio-Products, Woodland, CA) containing penicillin/streptomycin (Invitrogen Corporation) and supplemented with 1 ng/ml recombinant human IL-3. FLT3 point mutations in BaF3 cells were generated by site-directed mutagenesis in the pBabe Neo vector containing wild-type FLT3 cDNA using the QuickChange Site-Directed Mutagenesis kit following the manufacturer's recommendations (Stratagene, La Jolla, CA, USA). Primers for each point mutation were designed using Primer 3 software. All primers were ordered through Invitrogen Corporation. Primer sequences are listed in Supplemental Table 1.

All mutations were confirmed by sequencing. Transfection was performed using the Nucleofector II from Amaxa Biosystems (Walkersville, MD, USA), after which cells were selected in 1 mg/ml G418 in the presence of IL-3 and analyzed for FLT3 expression using CD135-PE antibody on a Becton-Dickinson FACSCalibur flow cytometer (Becton-Dickinson, San Jose, CA, USA) using CellQuest software. Clones were subsequently obtained by limiting dilution and tested for their ability to grow without IL-3. BaF3/ITD cells were established from a patient sample as previously described. [60]

### Growth inhibition assay

Based on Trypan Blue exclusion, 2 × 10^4^ viable cells were seeded in quadruplicate in a 96-well microtiter plate in the presence or absence of inhibitor for 48 h. The effect of drug activity on cell viability was measured using the 3-(4,5-Dimethylthiazol-2-yl)-2,5-diphenyltetrazolium bromide (MTT) assay according to the manufacturer's instructions (Roche Applied Science, Indianapolis, IN, USA). Following treatment, cells were incubated for 4 h in MTT followed by dissolution in solubilization buffer overnight. Absorbance was measured at 570 nm on a Bio-Rad plate reader (Hercules, CA, USA). The concentration of drug that inhibited absorbance by 50% (IC_50_) was calculated using CalcuSyn software. Results shown are presented as means and are representative of at least three independent experiments.

### Immunoprecipitation and western blotting

Activated FLT3 expression was measured by performing immunoprecipitation, SDS-PAGE and Western blotting as previously described. [60] Specifically, 10^7^ cells were washed in medium and treated with inhibitor for 1 h followed by stimulation with 25 ng/ml of FLT3 ligand for 15 minutes where indicated. Cells were washed twice with ice-cold PBS and lysed in 300 µl of NP-40 lysis buffer (150 mM NaCl, 20 mM TrisHCl (pH7.4), 10% glycerol, 1% NP-40, 10 mM EDTA, 100 mM NaF) with 2 mM Na_3_VO_4_ and protease inhibitors (Complete Tablets, Roche, Mannheim, Germany) at 4°C for 30 m. Cell lysate was harvested by centrifugation at 13,000 × *g* for 30 m at 4°C. FLT3 S-18 antibody was incubated with 500 µg lysate overnight at 4°C followed by immunoprecipitation with protein A-agarose beads for 2 h. Beads were washed once in Tris buffered saline with 1% Tween-20 (TBST) and twice in TBS. Samples were boiled in sodium dodecyl sulfate (SDS) sample buffer prior to separating in a 8% SDS-polyacrylamide gel (SDS-PAGE). Proteins were transferred to a polyvinyl diflouride (PVDF) membrane (Millipore, Bedford, MA, USA) and detected by 4G10 phosphotyrosine antibody and anti-FLT3 S-18 antibody. FLT3 was visualized using the Odyssey Infra Red Imaging System from Li-Cor (Lincoln, NE, USA) using AlexaFluor 680 goat anti-mouse and AlexaFluor 800 goat anti-rabbit antibodies. Phosphorylation of the FLT3 downstream signaling mediators, STAT5, AKT and MAP kinase was investigated by loading 50 µg of whole cell lysate and probing with the indicated antibodies. The membrane was washed and incubated in goat anti-rabbit secondary antibody conjugated to horseradish peroxidase followed by enhanced chemiluminescence (ECL). The membrane was subsequently stripped in 200 mM glycine buffer, pH 2.5 with 0.1% Tween-20 and reprobed for STAT5, AKT and MAP kinase expression using the respective antibodies.

### Engraftment in BALB/c mice

BaF3 D835Y cells were transfected with the L_3_GFP plasmid (a gift from Dr. Linzhao Cheng of Johns Hopkins University) containing genes for luciferase and green fluorescent protein (GFP). Cells were sorted for GFP and CD135 expression on a FACS AriaII cytometer using FACSDiva software. For engraftment, 2 ×10^6^ cells were injected *via* tail vein into BALB/c mice (Jackson Laboratories, Bar Harbor, ME, USA) (day 0). Starting five days later, mice were treated with either 20 mg/kg of lestaurtinib twice daily by subcutaneous injection as previously described or with the lestaurtinib vehicle to serve as a control or 10 mg/kg sorafenib suspended in 30%(w/v) Cremophor EL, 30% (w/v) PEG 400, 10% ethanol, and 10% glucose (all Sigma-Aldrich), once daily by oral gavage. [36, 37] These drug doses have previously been demonstrated to be effective against FLT3/ITD in mice. Mice were imaged by intraperitoneal injection of luciferin (3 mg) and visualizing on an IVIS Spectrum imager (Caliper LifeSciences, Hopkinton, MA, USA) using Living Image software for analysis on day 5 following inoculation to monitor engraftment and on day 9 to assess drug effect. All animal procedures were conducted in accordance with the policy of the Johns Hopkins University School of Medicine Animal Care and Use Committee.

### Authorship

B.N. and A.B.W. designed experiments, performed research, analyzed data and wrote the manuscript; H.M. revised the manuscript; L.L. performed research; P.B. analyzed data; M.L. analyzed data; D.S. designed experiments, supervised the project, analyzed data and wrote the manuscript.

## SUPPLEMENTARY MATERIALS FIGURES AND TABLES


