# Supplementary material for: FLT3 activating mutations display differential sensitivity to multiple tyrosine kinase inhibitors
Source: Oncotarget. 2017 Jan 6;8(7):10931–44. doi: 10.18632/oncotarget.14539 (PMC5355235; doi:10.18632/oncotarget.14539)
Supplement: Supplementary file 1 [file oncotarget-08-10931-s001.pdf]

FLT3 activating mutations display differential sensitivity to multiple tyrosine kinase inhibitors

Bao Nguyen[1,*], Allen B. Williams[1,*], David J. Young[1,2], Hayley Ma[1], Li Li[1], Mark Levis[1], Patrick Brown[1,2] and Donald Small[1,2]

[1] Department of Oncology, Johns Hopkins University School of Medicine, Baltimore, MD, USA

[2] Department of Pediatrics Johns Hopkins University School of Medicine, Baltimore, MD, USA

[*] These authors have contributed equally to this work

***Correspondence to**: Donald Small, **email**: donsmall@jhmi.edu*

Keywords: acute myeloid leukemia, mutant FLT3, activation loop, tyrosine kinase inhibitors

***Received**: December 16, 2016* ***Accepted**: December 25, 2016* ***Published**: January 06, 2017*

Supplemental Table 1. Primer sequences

D835A-F, GTGACTTTGGATTGGCTCGAGCTATCATGAGTGATTCCAAC;

D835A-R, GTTGGAATCACTCATGATAGCTCGAGCCAATCCAAAGTCAC;

D835G-F, GTGACTTTGGATTGGCTCGAGGTATCATGAGTGATTCCAAC;

D835G-R, GTTGGAATCACTCATGATAGCTCGAGCCAATCCAAAGTCAC;

D835N-F, GTGACTTTGGATTGGCTCGAAATATCATGAGTGATTCCAAC;

D835N-R, GTTGGAATCACTCATGATATTTCGAGCCAATCCAAAGTCAC;

D835L+K-F, GTGACTTTGGATTGGCTCGACTTAAAATCATGAGTGATTCCAAC;

D835L+K-R, GTTGGAATCACTCATGATTTTAAGTCGAGCCAATCCAAAGTCAC;

D835Y-F, GTGACTTTGGATTGGCTCGATATATCATGAGTGATTCCAAC;

D835Y-R, GTTGGAATCACTCATGATATATCGAGCCAATCCAAAGTCAC;

I836L+D-F, GTGACTTTGGATTGGCTCGAGATCTCGACATGAGTGATTCCAAC;

I836L+D-R, GTTGGAATCACTCATGTCGAGATCTCGAGCCAATCCAAAGTCAC;

I836S-F, GTGACTTTGGATTGGCTCGAGATAGCATGAGTGATTCCAAC;

I836S-R, GTTGGAATCACTCATGCTATCTCGAGCCAATCCAAAGTCAC;

I836T-F, GTGACTTTGGATTGGCTCGAGATACCATGAGTGATTCCAAC;

I836T-R, GTTGGAATCACTCATGGTATCTCGAGCCAATCCAAAGTCAC;

840G+S-F, GATATCATGAGTGATTCCGGCAGCAACTATGTTGTCAGGGGCAAT;

840G+S-R, ATTGCCCCTGACAACATAGCCGTTGCTGGAATCACTCATGATATC.
